# Aspergillus flavus Keratitis After Penetrating Keratoplasty

**DOI:** 10.7759/cureus.88985

**Published:** 2025-07-29

**Authors:** Zoi Karagiannidou, Dimitrios Mikropoulos, Kostas G Boboridis

**Affiliations:** 1 Department of Ophthalmology, Aristotle University of Thessaloniki, Thessaloniki, GRC; 2 1st Department of Ophthalmology, School of Medicine, Aristotle University of Thessaloniki, Thessaloniki, GRC

**Keywords:** aspergillus flavus, corneal graft, corneal grafting, corneal transplantation, fungal keratitis, penetrating keratoplasty (pkp), therapeutic keratoplasty

## Abstract

Fungal keratitis is a rare but severe complication following penetrating keratoplasty (PKP). We report the clinical course, rapid deterioration, and management of a case of *Aspergillus flavus* keratitis occurring three months after PKP. A 69-year-old woman with Fuchs’ endothelial dystrophy developed pseudophakic bullous keratopathy following cataract surgery. She underwent an uneventful PKP. At two months postoperatively, the graft remained clear with a visual acuity of 2/10. Three months after keratoplasty, the patient presented with pain, redness, mucopurulent discharge, and decreased vision. Slit-lamp examination revealed graft haze with a central epithelial defect, stromal infiltrates, and striae that rapidly progressed into the adjacent recipient cornea. Corneal scrapings were obtained for direct microscopic examination and culture, which revealed septate fungal filaments identified as *Aspergillus flavus*. Both the donor corneoscleral rim and the recipient corneal tissue were cultured at the time of surgery, and all cultures returned negative for fungal growth. Considering the negative donor cultures, the delayed onset of infection, and the presence of a persistent epithelial defect, the infection was most consistent with a postoperative superinfection rather than a donor-transmitted source. Intensive topical voriconazole and amphotericin B, along with systemic voriconazole, were initiated. Antifungal susceptibility testing was not performed due to the urgent need to initiate empirical therapy following the identification of fungal filaments on direct microscopy and rapid clinical deterioration. Despite aggressive antifungal therapy, the infection progressed, leading to graft melting and corneal perforation. The patient underwent therapeutic PKP, open sky vitrectomy, and removal of the posterior chamber intraocular lens (PC-IOL). Following the removal of the PC-IOL intraoperatively, the patient remained aphakic. Postoperatively, there was no recurrence of infection, and best-corrected visual acuity (BCVA) stabilized at 6/10 with aphakic spectacle correction. This case highlights the virulence of *Aspergillus flavus* and the challenges in managing post-keratoplasty fungal keratitis, especially when predisposing factors such as persistent epithelial defect and corticosteroid use are present. Early diagnosis and prompt medical and surgical intervention are critical to preserving ocular integrity.

## Introduction

Fungal keratitis is a rare but serious sight-threatening complication following corneal transplantation. It may originate from contaminated donor tissue or intraoperative fungal exposure during keratoplasty procedures [1]. Additionally, infections may develop during the postoperative period [2]. Although fungi cannot penetrate an intact epithelium, the risk of infection increases significantly in the presence of an epithelial defect or delayed epithelial healing following keratoplasty. The use of broad-spectrum topical antibiotics may disrupt the normal ocular surface flora, creating an environment conducive to fungal growth and proliferation. Simultaneously, topical corticosteroids that are commonly used post transplantation to suppress immune responses inhibit host defenses and enhance fungal replication [3]. The risk is further elevated by the use of bandage soft contact lenses (BSCL), which have been associated with a higher incidence of fungal infections [4].

Among fungal pathogens, *Candida* species are the most frequently reported causes of keratitis following both lamellar and penetrating keratoplasty (PKP) [5,6]. Less commonly, organisms such as *Cladosporium*, *Cryptococcus*, and *Aspergillus* species have also been implicated [7-9]. While *Aspergillus* keratitis has been described after refractive procedures, reports of graft infections specifically caused by *Aspergillus flavus* remain rare [10-13]. Specific virulence factors of *Aspergillus flavus* include rapid sporulation, thermotolerance, and the production of aflatoxins. These features may contribute to its aggressive behavior in corneal infections compared to other *Aspergillus* species. Fungal keratitis remains a significant clinical challenge, particularly in developing countries. It is a major cause of blindness in Asia and represents the leading indication for corneal transplantation in China [14-16]. Compared to bacterial keratitis, fungal infections are typically more aggressive. At the advanced stage, fungal keratitis is poorly responsive to medical treatment, necessitating surgical intervention such as PKP [17]. Clinically, fungal keratitis can be distinguished from bacterial forms by features such as feathery-edged corneal ulcers, ring infiltrates, satellite lesions, and plaque formation [18]. In severe cases, fungal organisms may penetrate into the anterior chamber, resulting in anterior chamber involvement or endophthalmitis, conditions associated with significantly poorer visual outcomes [18].

This report presents a rare case of *Aspergillus flavus* keratitis in a patient with a persistent epithelial defect and continued use of topical corticosteroids following PKP.

## Case presentation

A 69-year-old immunocompetent woman presented with acute, painful vision loss in her right eye, accompanied by photophobia, redness, and mucopurulent discharge. She had no significant past medical history or systemic illnesses, nor was she currently taking any medications. Her ophthalmic history included bilateral Fuchs’ endothelial dystrophy and cataract surgery in the right eye two years earlier, which resulted in pseudophakic bullous keratopathy. Examination revealed coalescent guttata, polymegathism, loss of hexagonal endothelial cells, stromal edema, and bullae formation. Despite treatment with hypertonic sodium chloride 5% eye drops, she experienced pain from ruptured bullae. Visual acuity was reported as 1/10 in the right eye. Consequently, she underwent PKP in the affected eye.

**Figure 1:**
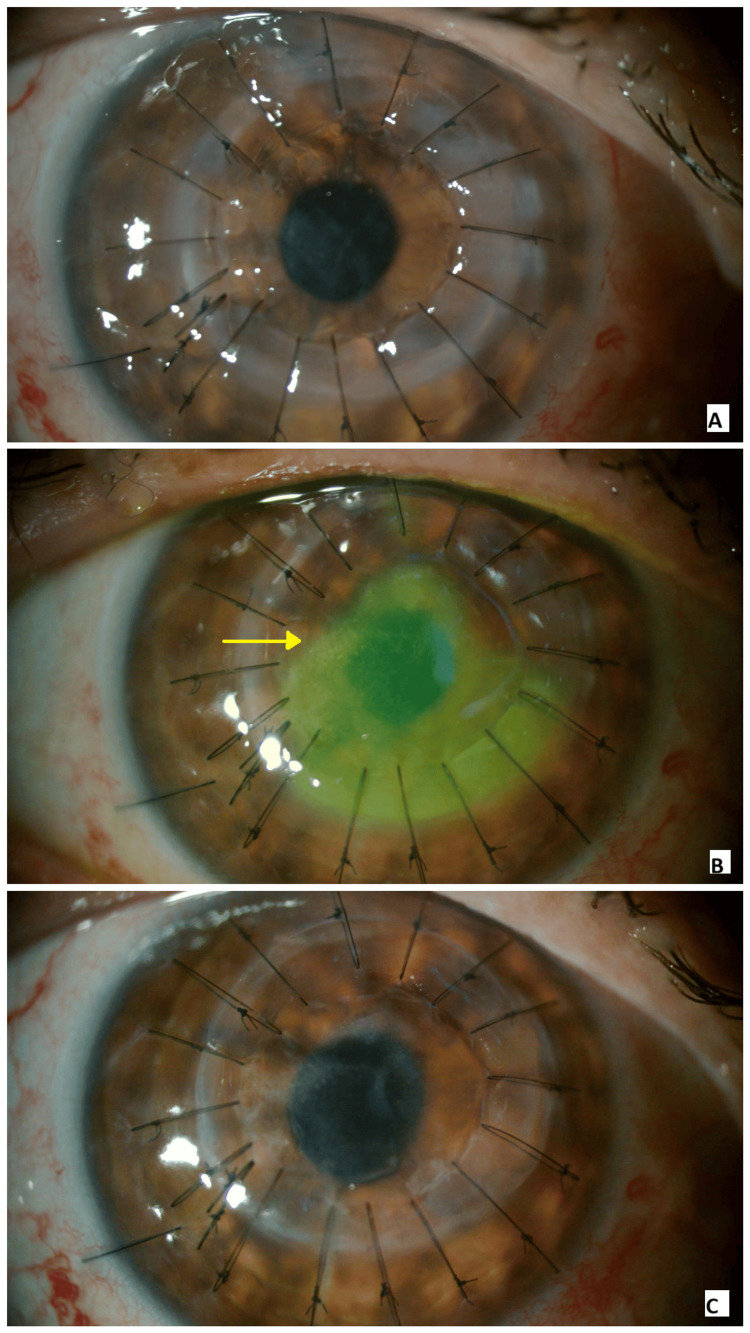
(A) On the first postoperative day, all sutures were intact, and the anterior chamber was well formed. (B) One week postoperatively, the sutures remained intact; however, a central epithelial defect was noted with fluorescein staining (yellow arrow), along with mild graft folds. (C) At two months postoperatively, the graft remained clear, all sutures were in place, and the anterior chamber was deep.

Surgical asepsis was ensured by scrubbing the periorbital skin with 10% povidone-iodine and instilling 5% povidone-iodine into the conjunctival fornix for three minutes before the procedure. The surgery proceeded uneventfully. A 7.25 mm trephine was used for the recipient cornea, and the donor cornea measured 0.25 mm larger. The graft was secured with 17 interrupted 10-0 nylon sutures, and an additional 9-0 nylon suture was placed at the seven o’clock position due to prior corneal trauma. At the end of the procedure, dexamethasone and amikacin were injected subconjunctivally. The donor cornea had been preserved in Optisol-GS (stored at 4°C for six days), which contains 2.5% chondroitin sulfate, 1% dextran, vitamins, and adenosine triphosphate (ATP) precursors. Cultures of both the recipient cornea and the donor corneoscleral rim were negative. On the first postoperative day, all sutures were intact, the anterior chamber was well formed, and there was no leakage (Figure 1A). The initial postoperative regimen included topical 0.1% dexamethasone four times daily, levofloxacin four times daily, and preservative-free artificial tears six times daily. While topical levofloxacin is necessary to prevent bacterial infection, we acknowledge that prolonged use of broad-spectrum antibiotics can disrupt the normal ocular surface flora and potentially increase susceptibility to fungal colonization. One week postoperatively, the sutures remained intact; however, a central epithelial defect was noted with fluorescein staining, along with mild graft folds (Figure 1B). Epithelial healing was closely monitored at each follow-up visit. To promote re-epithelialization, we administered preservative-free artificial tears and applied a BSCL. While the use of topical growth factors was considered, initial healing progressed adequately without additional interventions. While the use of a BSCL in the presence of an epithelial defect can increase the risk of microbial colonization, in our case, the BSCL was applied under sterile conditions, and the patient was closely monitored with regular follow-up visits. The lens was promptly removed upon completion of re-epithelialization. The postoperative course was initially unremarkable. The epithelial defect and stromal edema gradually resolved. At one and two months postoperatively, the graft remained clear, all sutures were in place, the anterior chamber was deep, and visual acuity had improved to 2/10 in the right eye (Figure 1C).

**Figure 2:**
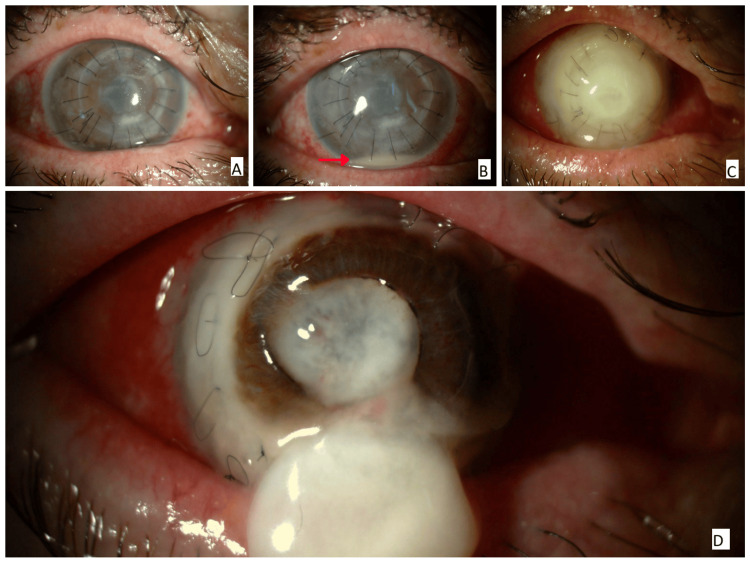
(A) Graft haze, a central epithelial defect, stromal infiltrates, and striae. (B) The graft and adjacent recipient cornea became opaque, and hypopyon developed in the anterior chamber (red arrow). (C) The cornea became progressively thinner, whiter, and opaque. Anterior chamber details were no longer visible, and the sutures were loosened. (D) The graft began to melt and eventually perforated.

Three months after keratoplasty, the patient presented with acute, painful vision loss in the right eye, along with photophobia, redness, and mucopurulent discharge. Visual acuity had deteriorated to counting fingers (CF) at 20 cm. Slit-lamp examination revealed diffuse conjunctival injection, graft haze, a central epithelial defect with fluorescein staining, stromal infiltrates, and striae (Figure 2A). Corneal scrapings were taken for direct microscopy and culture. The patient was initially treated with topical dexamethasone, levofloxacin, and lubricants. At presentation, the patient had a clear central epithelial defect, graft edema, and stromal haze without evident stromal infiltrates, satellite lesions, or anterior chamber reaction, features that were more suggestive of immune-mediated graft rejection. There was also no pain out of proportion to clinical findings or raised suspicion of infection at that time. Therefore, the initial impression favored graft rejection. Suspecting graft rejection, our patient was started on oral methylprednisolone at a dose of 16 mg three times daily. However, the clinical condition deteriorated rapidly over a few days. The graft and adjacent recipient cornea became opaque, and hypopyon developed in the anterior chamber (Figure 2B). Visual acuity declined further to hand motion (HM) only. Given the high suspicion of fungal keratitis, all corticosteroids were discontinued. Scrapings from the ulcer bed and edges were sent for Gram and Giemsa staining, 10% potassium hydroxide (KOH) mount, and culture on various media (blood agar, chocolate agar, brain-heart infusion broth, Sabouraud dextrose agar, thioglycollate broth, and non-nutrient agar with an overlay of live *Escherichia coli*). Features more suggestive of fungal keratitis include feathery-edged stromal infiltrates, satellite lesions, dry texture, plaque formation, and gradual progression. In our case, these features developed over time. The initial Gram stain was negative for bacteria, but the 10% KOH mount and Giemsa stain revealed septate fungal filaments, which raised a strong suspicion for a fungal etiology and guided further management. While septate hyphae seen on smear suggested a filamentous fungus, *Aspergillus* was not specifically identified at that stage. Given the high clinical suspicion for filamentous fungal keratitis, we initiated broad-spectrum antifungal therapy with both topical voriconazole and amphotericin B to cover both *Aspergillus* and *Fusarium* species. Systemic voriconazole was also chosen because of its effectiveness against filamentous fungi and good ocular penetration. The identification of *Aspergillus flavus* was later confirmed by culture. The patient was started on intensive antifungal therapy, including fortified voriconazole 1% and amphotericin B 0.15% eye drops administered hourly, along with intravenous voriconazole 400 mg twice daily on the first day (loading dose), followed by 250 mg twice daily. Baseline and biweekly liver function tests were performed throughout the course of treatment. Antifungal susceptibility testing was performed using standard microdilution techniques, according to the Clinical and Laboratory Standards Institute (CLSI) guidelines. The *Aspergillus flavus* isolate was found to be susceptible to voriconazole, with a minimum inhibitory concentration (MIC) of 0.5 µg/mL. Amphotericin B demonstrated an MIC of 1 µg/mL, indicating intermediate susceptibility. Despite aggressive medical management, the corneal condition continued to worsen. The cornea became progressively thinner, whiter, and opaque. Anterior chamber details were no longer visible, sutures were loosened, the graft began to melt, and eventually perforated (Figures 2C, 2D).

The patient underwent a repeat PKP under general anesthesia. Standard preoperative antisepsis was performed, including scrubbing of the periorbital skin with 10% povidone-iodine solution and instillation of 5% povidone-iodine into the conjunctival fornix for three minutes. Intraoperatively, the anterior chamber was thoroughly irrigated. An anterior vitrectomy was performed, followed by removal of the intraocular lens (IOL) and an open-sky posterior vitrectomy. A 9.0 mm donor cornea was used. After reformation of the anterior chamber with an ophthalmic viscoelastic device, the infected recipient graft was excised, and the new donor graft was secured with 16 interrupted 10-0 nylon sutures. At the conclusion of the surgery, 0.1 mL of intracameral voriconazole (50 μg/0.1 mL) was administered. The donor cornea had been preserved in Optisol-GS for five days prior to transplantation. Cultures of both the excised recipient cornea and the donor corneoscleral rim were taken; both were negative.

**Figure 3:**
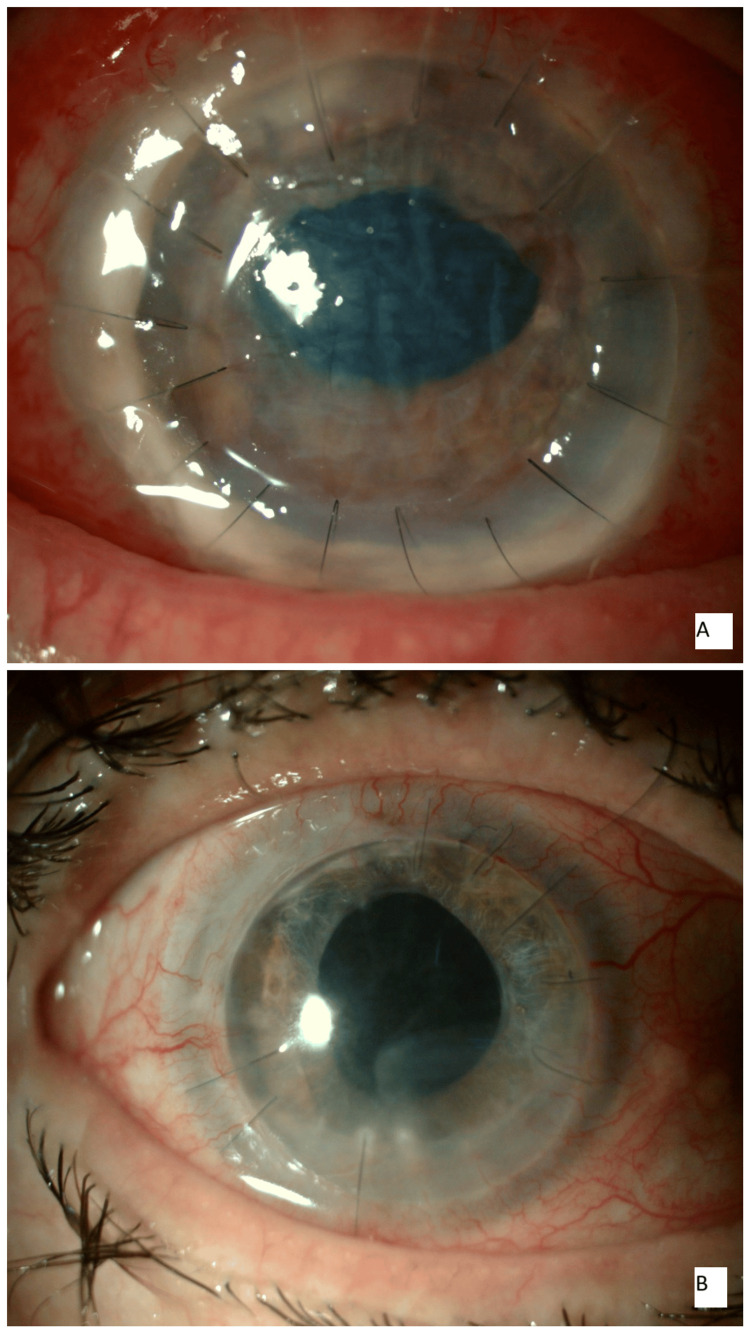
(A) On postoperative day one, an intact graft with epithelial defect, mild corneal edema, and striae was noted; the anterior chamber was well-formed, with intact 16 sutures. (B) At the two-month follow-up, the graft appeared clear with mild peripheral neovascularization, and the anterior chamber was deep and quiet. Intact eight sutures (eight sutures were removed due to localized suture-related vascularization).

Postoperatively, the patient was managed with intensive antifungal therapy, including topical amphotericin B 0.15% and voriconazole 1% administered hourly. Broad-spectrum topical antibiotics, including ceftazidime, vancomycin, and tobramycin, were added for prophylaxis against secondary bacterial infections. Preservative-free artificial tears were applied six times daily. Topical dexamethasone was initially withheld for two weeks and then reintroduced at a frequency of four times daily. Intravenous voriconazole 250 mg twice daily was also continued. On postoperative day one, slit-lamp examination showed an intact graft with epithelial defect, mild corneal edema, and striae; the anterior chamber was well-formed (Figure 3A). The initial postoperative course was uneventful, with gradual resolution of the epithelial defect and improvement in stromal edema and graft striae. By the end of the first postoperative week, visual acuity had improved to counting fingers (CF) at one meter. By the end of the third week, the best-corrected visual acuity (BCVA) reached 2/10. Suture management was performed as indicated; two loose sutures were removed at one month postoperatively, followed by the removal of six additional sutures by the end of the second month. Two sutures were removed within the first postoperative month due to localized suture-related vascularization and irritation, which posed a risk to graft clarity and healing. Early suture removal in this context was deemed necessary to minimize these complications and promote graft survival. The timing was carefully balanced to reduce the risk of wound dehiscence. At the two-month follow-up, BCVA remained stable at 6/10 with aphakic spectacle correction. The graft appeared clear with mild peripheral neovascularization, the anterior chamber was deep and quiet, intraocular pressure was within normal limits, and fundoscopy findings were unremarkable (Figure 3B). Mild peripheral graft neovascularization was observed and closely monitored through regular slit-lamp examinations, along with assessments of intraocular pressure and endothelial cell health. Anti-VEGF therapy was not deemed necessary at this stage but remains a consideration if neovascularization progresses. Antifungal therapy was gradually tapered over the following 10 weeks. Intravenous voriconazole was switched to oral administration (250 mg twice daily) three weeks postoperatively to complete the treatment course.

## Discussion

We report a rare case of *Aspergillus flavus* keratitis following PKP, highlighting the complexity and therapeutic challenges associated with fungal infections of corneal grafts. Unlike bacterial keratitis, which typically responds well to antimicrobial therapy, fungal keratitis is more difficult to manage due to the limited efficacy of available antifungal agents. Poor ocular bioavailability, potential toxicity, and reduced solubility of many antifungal formulations significantly hinder treatment success [19-21].

Post-keratoplasty fungal keratitis is most commonly caused by *Candida* species, particularly following both lamellar and penetrating procedures [1,2]. *Candida* keratitis typically presents within the first two weeks postoperatively and is frequently associated with contaminated donor tissue [3-5]. Two main sources of fungal graft infections have been proposed, each with distinct clinical features. Donor-derived infections usually involve the posterior stroma and manifest approximately three to four months postoperatively. Conversely, superinfections arising from epithelial defects or suture-related complications typically occur earlier and involve the superficial cornea. In our patient, cultures of both the donor corneoscleral rim and storage medium were negative, making a donor-derived infection less likely. However, contamination during eye bank handling or surgery cannot be entirely excluded. Environmental sources such as BSCL, storage solutions, and periocular flora were considered potential contributors to postoperative superinfection. Although cultures were not obtained from the lens or periocular area retrospectively, strict aseptic technique was adhered to during lens application and handling. Given the negative donor cultures and the presence of a persistent epithelial defect, the infection was most likely a postoperative superinfection. The ulcer developed in the area of previously delayed re-epithelialization, a region particularly susceptible to microbial invasion. Additionally, the use of topical corticosteroids facilitated the rapid disease progression by suppressing the local immune defenses [4-7]. The aggressive clinical course may also reflect the high virulence of *Aspergillus flavus*, which ranks second only to *Aspergillus fumigatus* in pathogenic potential. Specific virulence factors of *Aspergillus flavus* include its capacity for rapid sporulation, thermotolerance, and production of aflatoxins, potent mycotoxins known to cause tissue necrosis and immune modulation. Additionally, *Aspergillus flavus* exhibits a pronounced ability to adhere to and invade corneal tissue, often more aggressively than other *Aspergillus* species. These properties contribute to a more fulminant clinical course, including early stromal necrosis and graft melting, even in the absence of systemic immunosuppression. *Aspergillus flavus* is a cause of keratitis, sinusitis, cutaneous infections, and invasive fungal disease, and accounts for up to 80% of *Aspergillus*-related keratitis cases [13,14]. Established risk factors include ocular trauma (especially involving plant matter) and recent corneal refractive or cataract surgery [10-13]. In our case, the combination of prior ocular surgery, persistent epithelial defect, topical corticosteroid use, and therapeutic contact lens wear created a favorable environment for fungal infection. At initial presentation, the clinical features were more consistent with graft rejection rather than infection, and there were no clear signs suggestive of fungal keratitis. Therefore, systemic corticosteroids were appropriately initiated as first-line treatment. Given the absence of fungal indicators at that time, antifungal therapy was not started empirically to avoid unnecessary toxicity and resistance. However, once fungal keratitis was suspected and confirmed, antifungal treatment was promptly initiated. In hindsight, combination therapy at presentation may carry risks and was not justified based on the clinical picture.

The Infectious Diseases Society of America (IDSA) recommends intravenous voriconazole (3-4 mg/kg twice daily) for fungal infections, including *Candida* endophthalmitis, citing its excellent intraocular penetration [20,21]. Intravitreal voriconazole has been successfully used in cases of *Aspergillus* endophthalmitis, as well [22,23]. Moreover, systemic and topical voriconazole proved effective. In vitro studies show that *Aspergillus flavus* is most sensitive to natamycin, followed by amphotericin B and terbinafine, with limited susceptibility to ketoconazole [20-26]. However, the clinical utility of topical amphotericin B is constrained by poor corneal penetration and ocular surface toxicity, especially at higher doses or with prolonged use [20]. Furthermore, resistance to amphotericin B and itraconazole has been increasingly reported. Due to rapid clinical deterioration, urgent repeat keratoplasty was necessary. The patient responded favorably to a combination of surgical intervention and intensive antifungal therapy, which included intraoperative intracameral antifungal injection (voriconazole), systemic and topical voriconazole, and topical amphotericin B [25,26]. Antifungal therapy was continued for a total duration of 10 weeks, with intravenous voriconazole transitioned to oral administration after three weeks. The treatment was tapered according to clinical improvement. No long-term complications, including endothelial rejection or secondary glaucoma, were observed during the three-month follow-up period.

## Conclusions

This case underscores the virulence of *Aspergillus flavus* and the challenges in managing post-keratoplasty fungal keratitis, especially when predisposing factors such as persistent epithelial defect and corticosteroid use are present. Early clinical suspicion, prompt microbiological confirmation, and timely surgical and pharmacologic interventions are crucial for managing fungal infections of the corneal graft. Early clinical signs that raised suspicion of fungal keratitis included a persistent non-healing epithelial defect, feathery-edged stromal infiltrates, and rapid graft deterioration despite ongoing corticosteroid therapy. These findings prompted immediate microbiological investigation. In our experience, antifungal therapy with systemic and topical voriconazole and topical amphotericin B was effective in the treatment of *Aspergillus flavus* infection. The patient underwent biweekly liver function tests and regular ocular examinations throughout the antifungal treatment course. No systemic or ocular toxicity was observed. The favorable outcome achieved, in spite of the devastating nature of the infection, is attributed to rapid diagnosis and timely medical and surgical intervention. In our case, the interval from symptom onset to surgical intervention was approximately 10 days. This delay, unfortunately, exceeded the recommended window for optimal outcomes in fungal keratitis, which is generally within the first five to seven days of symptom onset. The delay was primarily due to an initial misdiagnosis of immunologic graft rejection, which temporarily masked the underlying fungal infection. This case underscores the importance of early recognition and prompt intervention in fungal keratitis to prevent irreversible corneal damage and potential graft failure.
